# Arecoline induces HA22T/VGH hepatoma cells to undergo anoikis - involvement of STAT3 and RhoA activation

**DOI:** 10.1186/1476-4598-9-126

**Published:** 2010-05-28

**Authors:** Hsiao-Ling Cheng, Shu-Jem Su, Li-Wen Huang, Bau-Shan Hsieh, Yu-Chen Hu, Thu-Ching Hung, Kee-Lung Chang

**Affiliations:** 1 Graduate Institute of Medicine, College of Medicine, Kaohsiung Medical University, Kaohsiung 80708, Taiwan; 2 Bachelor Degree Program of Health Beauty, Department of Medical Technology, School of Medicine and Health Sciences, FooYin University, Kaohsiung 83101, Taiwan; 3 Department of Medical Laboratory Science and Biotechnology, Kaohsiung Medical University, Kaohsiung 80708, Taiwan; 4 Department of Biochemistry, Faculty of Medicine, College of Medicine, Kaohsiung Medical University, Kaohsiung 80708, Taiwan

## Abstract

**Background:**

Our previous study showed that, in basal cell carcinoma cells, arecoline reduces levels of the tumor cell survival factor interleukin-6 (IL-6), increases levels of tumor suppressor factor p53, and elicits cell cycle arrest, followed by apoptosis. In preliminarily studies, we observed that arecoline induces detachment of the human-derived hepatoma cell line HA22T/VGH from the extracellular matrix. In the present study, we explored the fate of the detached HA22T/VGH cells and investigated the underlying mechanism.

**Methods:**

HA22T/VGH cells or primary cultured rat hepatocytes were treated with arecoline, then changes in morphology, viability, apoptosis, and the expression of surface β1-integrin, apoptosis-related proteins, and IL-6 were examined. Furthermore, activation of the signal transducer and activator of transcription 3 (STAT3) pathway and the RhoA/Rock signaling pathway, including p190RhoGAP and Src homology-2 domain-containing phosphatase SHP2, was examined.

**Results:**

A low concentration of arecoline (≤ 100 μg/ml) caused cytoskeletal changes in HA22T/VGH cells, but not hepatocytes, and this was accompanied by decreased β1-integrin expression and followed by apoptosis, indicating that HA22T/VGH cells undergo anoikis after arecoline treatment. IL-6 expression and phosphorylation of STAT3, which provides protection against anoikis, were inhibited and levels of downstream signaling proteins, including Bcl-X_L _and Bcl-2, were decreased, while Bax expression, mitochondrial cytochrome c release, and caspase-3 activity were increased. In addition, phosphorylation/activation of p190RhoGAP, a RhoA inhibitor, and of its upstream regulator, SHP2, was inhibited by arecoline treatment, while Rho/Rock activation was increased. Addition of the RhoA inhibitor attenuated the effects of arecoline.

**Conclusions:**

This study demonstrated that arecoline induces anoikis of HA22T/VGH cells involving inhibition of STAT3 and increased RhoA/Rock activation and that the STAT3 and RhoA/Rock signaling pathways are connected.

## Background

Arecoline has been suggested as a possible cognition enhancer in Alzheimer's type dementia [1,2]. Recent studies have shown that it decreases interleukin-6 (IL-6) production in keratinocytes and KB cancer cells [3,4]. In addition, Chang *et al*. [3] reported that arecoline elicits cell cycle deregulation in KB cancer cells. Moreover, our previous study [Chang *et al*.: Arecoline decreases interleukin-6 production and induces apoptosis and cell cycle arrest in human basal cell carcinoma cells (BCC/KMC), submitted] showed that, in basal cell carcinoma cells, arecoline reduces levels of the tumor cell survival factor IL-6, increases levels of the tumor suppressor factor p53, and elicits cell cycle arrest, followed by apoptosis, showing that arecoline interferes with cancer cell cycle progression. Our preliminary data showed that arecoline induces detachment of the hepatoma cell line HA22T/VGH from the extracellular matrix (ECM).

Adherence of epithelial cells to the ECM is important for cell growth and survival and detachment from the ECM induces cell apoptosis, known as anoikis [5,6]. The expression of certain oncogenes, such as activation of signal transducer and activator of transcription 3 (STAT3) [7], phosphatidylinositol 3-kinase (PI3K)/Akt [8], and Src [8], provides anchorage-independent growth ability and protection against anoikis, and this protection is thought to be critical during tumorigenesis.

The small GTPase RhoA has emerged as a pivotal control point through which cells sense changes in ECM mechanics and cytoskeletal organization and translate the 'cell shape signal' to downstream effectors that mediate these behaviors [8]. RhoA activity can be suppressed by any one of a variety of different RhoGAP proteins. p190RhoGAP has been shown to be phosphorylated by Src tyrosine kinase when cells first attach to the ECM substrate and integrin receptors become ligated, allowing p190RhoGAP to exert its RhoGAP activity and leading to inactivation of RhoA [9,10]. Cell detachment and rounding in mitosis have also been reported to inhibit p190RhoGAP activity and increase RhoA activity [11].

Src homology-2 domain-containing phosphatases (SHPs) are a small, highly conserved subfamily of protein-tyrosine phosphatases, members of which are present in both vertebrates and invertebrates. In most receptor tyrosine kinase signaling pathways, SHP2 is required for full activation [12]. SHP2 has been reported to play an essential role in integrin signaling, and dominant-negative mutants of SHP2 inhibit integrin-stimulated focal adhesion and stress fiber turnover, cell spreading, and proliferation [12].

In the present study, we explored the fate of the HA22T/VGH cells detached by the action of arecoline and investigated the underlying mechanisms of this detachment. Cytokine IL-6 expression and activation of its downstream effector STAT3 and expression and activation of RhoA/Rock, p190RhoGAP, and SHP2 were also examined. Our results showed that arecoline induces anoikis in HA22T/VGH cells by inhibiting the activation of STAT3, SHP2 and p190RhoGAP and enhancing the activation of RhoA/Rock.

## Results

### Arecoline induces cell detachment, followed by apoptosis

**Figure 1:**
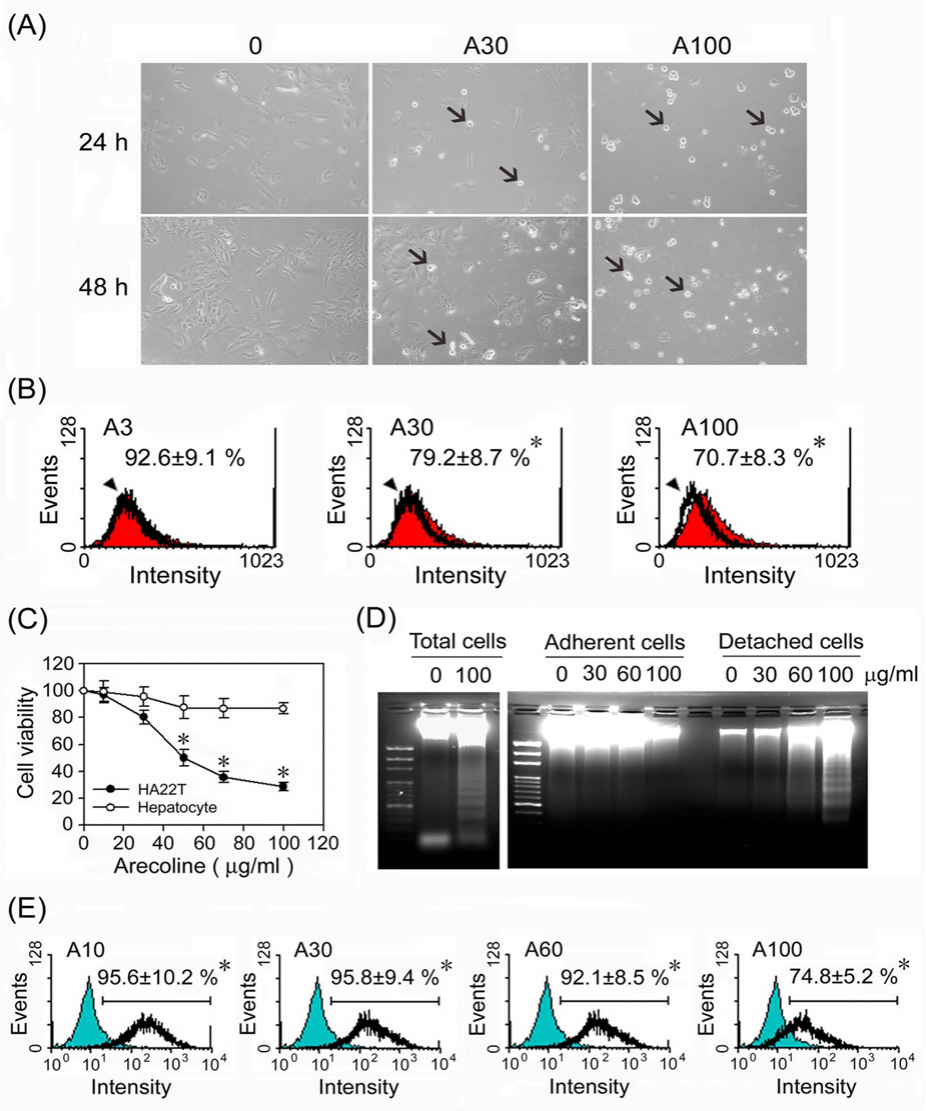
**Arecoline induces detachment of HA22T/VGH cells, followed by apoptosis**. (A) HA22T/VGH cells were treated with 0 (left), 30 (center), or 100 (right) μg/ml of arecoline for 24 h or 48 h, then cell morphology was observed under a phase-contrast microscopy at 200× magnification. The black arrows indicate detached cells. (B) After treatment of arecoline for 24 h, β1-integrin expression was measured by flow cytometric analysis using RPE-conjugated antibody. The histogram of red filled area is the untreated control and the black lines the treated groups. The values shown are the mean fluorescence intensity as a percentage of the untreated control value. (C) HA22T/VGH cells or primary normal rat hepatocytes were treated with the indicated concentration of arecoline for 72 h, then viable cells were counted using Trypan blue and the results expressed as a percentage of the untreated control value. (D) After treatment of arecoline for 72 h, the cells were harvested all together or the detached and adherent cells separately and genomic DNA extracted and analyzed on an agarose gel for DNA fragmentation. The left panel shows the total cells and the right panel adherent and detached cells separately. (E) TUNEL staining of the detached cells detected by flow cytometric analysis. The green filled area is the untreated control and the black lines the treated groups. The values shown are the percentage of TUNEL-positive cells in the detached cells. All data are the mean ± S.D. for three independent experiments. *: p < 0.05 as compared to the untreated control.

As in our preliminary study, some HA22T/VGH cells became detached after 24 h of treatment with 30 or 100 μg/ml of arecoline, and more became detached after 48 h of treatment (Fig. 1A). This arecoline-induced cell detachment was accompanied by decreased expression of the cell surface adhesion molecule β1-integrin (Fig. 1B). To clarify whether this detachment was due to cell cycle progression, we examined the distribution of cell cycle phases and found there was no difference with or without arecoline treatment (data not shown). Having excluded cell cycle progression, we explored the fate of these detached cells and examined the effects of arecoline on normal rat hepatocytes. Interestingly, no detachment of normal hepatocytes was seen with arecoline treatment. After 72 h of arecoline treatment, the viability of normal hepatocytes was not significantly changed, whereas that of HA22T/VGH cells decreased in a dose-dependent manner (Fig. 1C). In addition, DNA fragmentation was seen in arecoline-treated HA22T/VGH cells and was restricted to the detached cells (Fig. 1D). As shown in Fig. 1E, more than 90% of the detached cells were positive for TUNEL staining over the concentration range of 10 μg/ml to 60 μg/ml arecoline, while only 74% were positive at the concentration of 100 μg/ml, which could be explained by the fact that some of the detached cells had died. These results demonstrate that arecoline induces HA22T/VGH detachment, followed by apoptosis.

### Expression of apoptosis-related proteins and caspase activity

**Figure 2:**
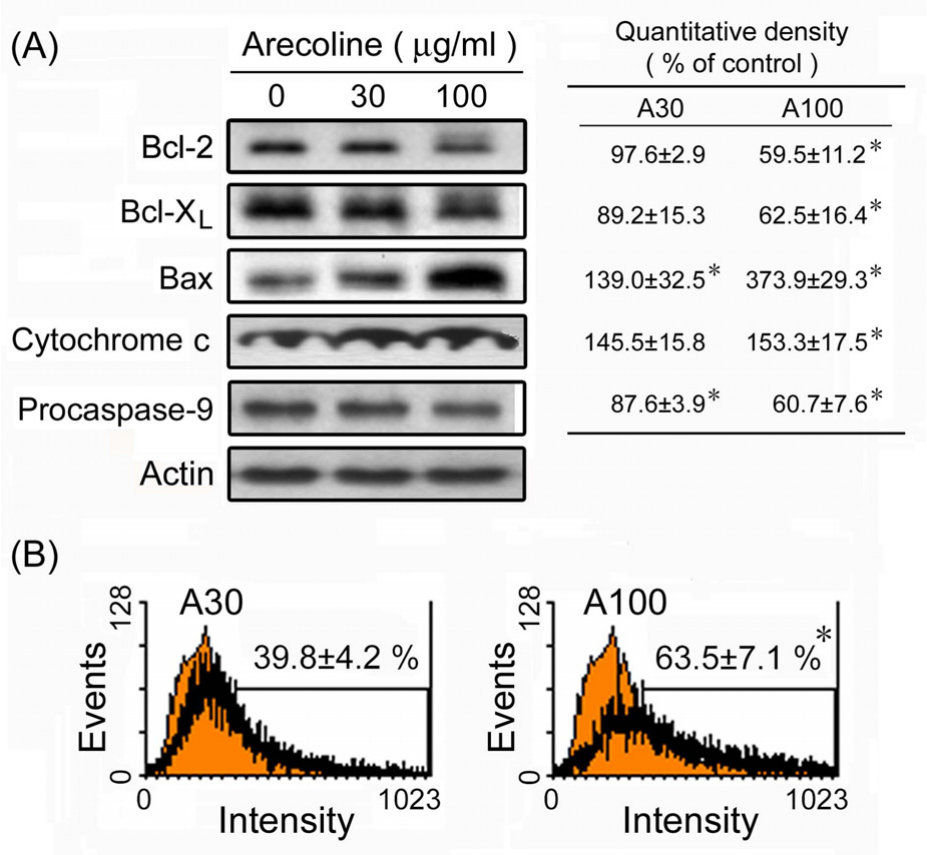
**Arecoline alters the expression of apoptosis-related proteins and caspase activity in HA22T/VGH cells**. (A) HA22T/VGH cells were treated with 0, 30, or 100 μg/ml of arecoline for 24 h, then the cells were harvested and proteins extracted and used for Western blotting for Bcl-2, Bcl-X_L_, Bax, cytochrome c, or procaspase-9. β-actin was used as the internal control. The values shown are the quantitative density analysis expressed as the relative density compared to that in untreated cells (control), taken as 100%. The results are expressed as the mean ± S.D. for three separate experiments. (B) Caspase-3 activity was detected using RPE-conjugated anti-active caspase-3 antibody by flow cytometric analysis. The values shown are the percentage of cells with active caspase-3 and are the mean ± S.D. of three independent experiments. The red filled area is the untreated control and the black lines the treated groups. *: p < 0.05 as compared to the untreated control.

To determine whether this arecoline-induced apoptosis was associated with altered expression of apoptosis-regulated proteins, HA22T/VGH cells were treated for 24 h with 30 or 100 μg/ml of arecoline. At 100 μg/ml of arecoline, Western blots showed a significant decrease in Bcl-2, Bcl-X_L_, and procaspase-9 levels and a significant increase in Bax levels and cytochrome c release (Fig. 2A). Members of the caspase family are expressed in cells as inactive procaspases, which are activated during apoptosis. As shown in Fig. 2B, treatment of HA22T/VGH cells for 24 h with 100 μg/ml of arecoline resulted in a marked increase in active caspase-3, as shown by flow cytometry using an antibody against active caspase-3. These results show that arecoline induces apoptosis by caspase-3 activation and a reduction in expression of anti-apoptotic proteins.

### Arecoline decreases IL-6 expression and STAT3 activation

**Figure 3:**
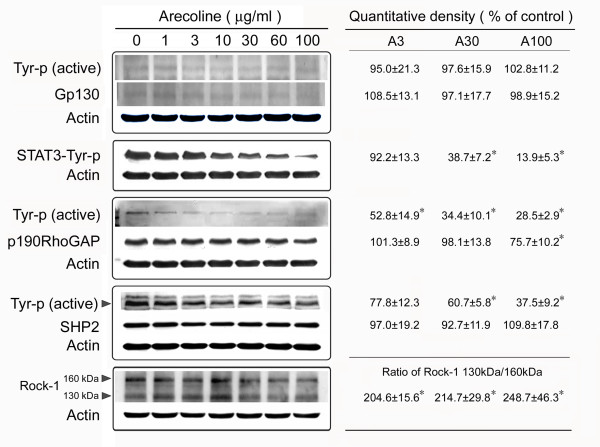
**Arecoline inhibits activation of STAT3, p190RhoGAP, and SHP2 and increases Rock-1 cleavage in HA22T/VGH cells**. HA22T/VGH cells were treated with 0-100 μg/ml of arecoline for 24 h, then the cells were harvested and proteins extracted and used for Western blotting with antibodies against Gp130, phospho-Tyr705-STAT3 (STAT3-Tyr-p), p190RhoGAP, SHP2, or Rock-1; the Gp130, p190RhoGAP, and SHP2 blots were then stripped and re-blotted using antibodies against phospho-Tyr (Tyr-p). β-actin was used as the internal control. The values shown are the quantitative density analysis expressed as the relative density compared to that in untreated cells (control), taken as 100%. The results are expressed as the mean ± S.D. for three separate experiments. *: p < 0.05 as compared to the untreated control.

Several studies have reported that anti-apoptotic genes are regulated by IL-6 and STAT3 [13,14]. Moreover, in the liver, STAT3 is mainly activated by IL-6 and related cytokines and promotes anchorage-independent growth [15-17]. To examine whether arecoline had an effect on IL-6 and/or STAT3 levels, HA22T/VGH cells were treated with 1-100 μg/ml of arecoline for 24 h, then IL-6 mRNA levels were measured by quantitative real-time PCR and levels of gp130 (an IL-6 signal transducing receptor component) and Tyr705 phosphorylated STAT3 (the active form of STAT3) were assayed by Western blotting. Table 1 shows that arecoline at concentrations of 30-100 μg/ml markedly decreased IL-6 expression, while Fig. 3 shows that levels of gp130 or activated gp130 were not altered by arecoline, while Tyr705 phosphorylation of STAT3 was markedly inhibited.

**Table 1:** Relative quantitation of IL-6 expression in HA22T/VGH after arecoline treatment by quantitative real-time PCR method.

Arecoline (μg/ml)	IL-6	GAPDH	ΔCt^a^	ΔΔCt^b^	related to control^c^
**0**	28.70 ± 0.09	17.34 ± 0.07	11.36 ± 0.11	0 ± 0.11	1.00 (0.92-1.08)
**3**	28.67 ± 0.11	17.28 ± 0.06	11.39 ± 0.13	0.03 ± 0.13	0.98 (0.90-1.07)
**30**	29.11 ± 0.06	17.23 ± 0.08	11.88 ± 0.10	0.52 ± 0.10	0.70* (0.65-0.75)
**100**	31.54 ± 0.08	17.67 ± 0.03	13.87 ± 0.09	2.51 ± 0.09	0.18^#^ (0.17-0.19)

^a^: The ΔCt value is determined by subtracting the average GAPDH Ct value from the average IL-6 Ct value. All data are the mean ± standard deviation. ^b^: The ΔΔCt value is determined by subtracting the control ΔCt value from the treatment ΔCt value. ^c^: The range given for IL-6 relative to control is determined by evaluating the expression: 2^(-ΔΔCt)^ with ΔΔCt+S.D. and ΔΔCt-S.D., where S.D. = the standard deviation of the ΔΔCt value. *: p < 0.05; #: p < 0.001 as compared to the untreated control.

### Arecoline inhibits p190RhoGAP and SHP2 activation and increases Rho-associated kinase

p190RhoGAP is phosphorylated by Src tyrosine kinase, allowing it to exert its RhoGAP activity, leading to inactivation of RhoA [9,10]. Fig. 3 shows that, after 24 h of arecoline treatment, p190RhoGAP levels were significantly decreased at the concentration of 100 μg/ml of arecoline, while levels of the active phosphorylated p190RhoGAP were decreased at arecoline concentrations higher than 3 μg/ml. Src homology-2 domain-containing phosphatases (SHPs) are present in vertebrates and invertebrates. SHP2 plays an essential role in integrin signaling [12] upstream of RhoA to regulate its activity [18]. As shown in Fig. 3, after 24 h of arecoline treatment, the amount of SHP2 was not changed, but the amount of active phosphorylated SHP2 was decreased at arecoline concentrations higher than 30 μg/ml. Furthermore, Rho-associated kinase-1 (Rock-1) was cleaved to generate an active cleaved form at arecoline concentrations higher than 3 μg/ml. The decreased activation of p190RhoGAP and SHP2 might result in enhanced RhoA activity and activity of its effector, Rock.

### Arecoline stimulates actin stress fiber formation

**Figure 4:**
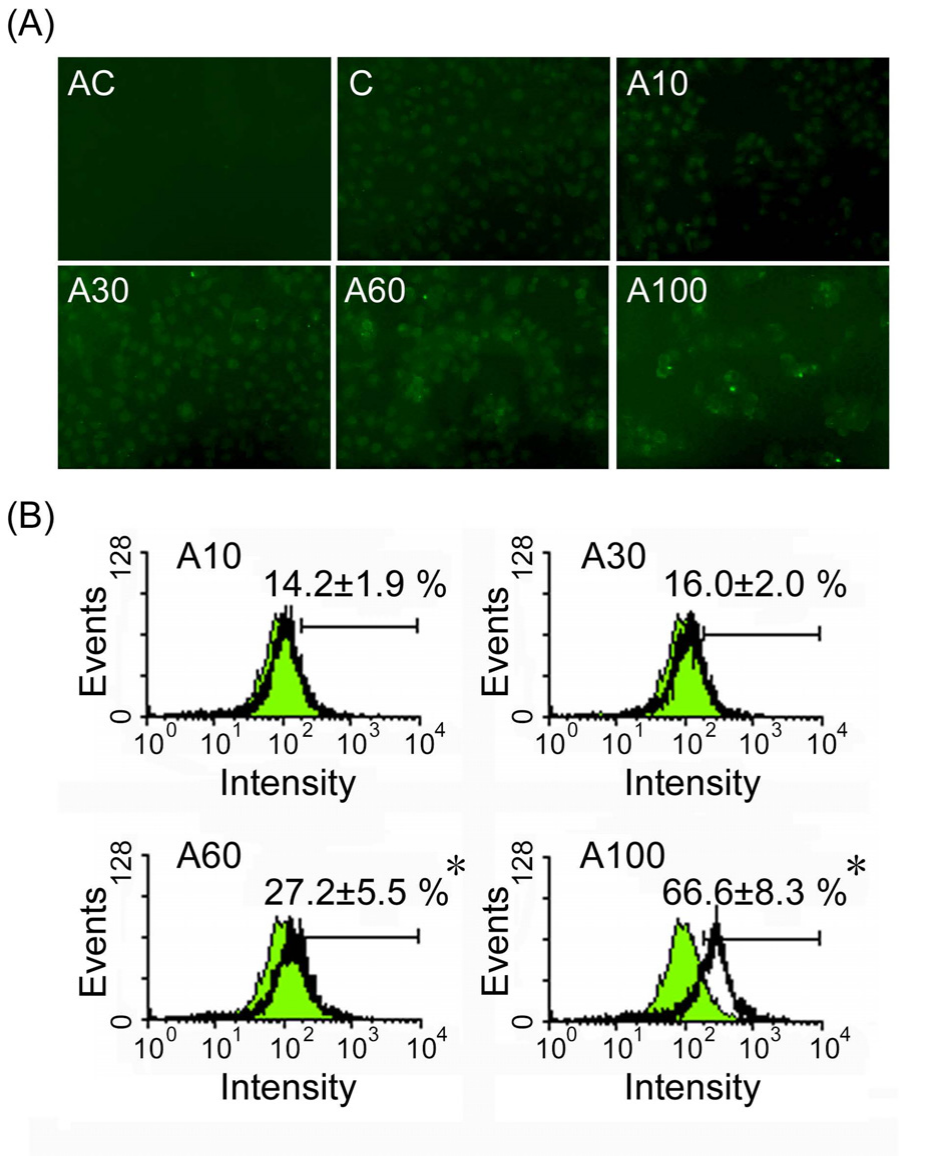
**Arecoline increases actin stress fiber formation in HA22T/VGH cells**. HA22T/VGH cells were treated with 0, 10, 30, 60, or 100 μg/ml of arecoline for 24 h, then were fixed with paraformaldehyde for actin labeling and observation by fluorescence microscopy (A) or flow cytometry (B). In A, panel AC is untreated cells incubated with second antibody alone, and C is the untreated control group incubated with primary and secondary antibodies. The values shown in B are the percentage of actin staining-cells and are the mean ± S.D. for three independent experiments. The green filled area is the untreated control and those delimited by the black lines the treated groups. *: p < 0.05 as compared to the untreated control.

RhoA induces assembly of actin-myosin filaments through activation of Rock kinase [19]. Since arecoline was found to increase the amount of active Rock, we examined whether it also stimulated actin stress fiber formation. After treatment with arecoline for 24 h, actin stress fiber formation was increased (Fig. 4A) and was increased by almost 6-fold at the concentration of 100 μg/ml arecoline compared to the control (Fig 4B).

### A RhoA inhibitor attenuates the effects of arecoline

**Figure 5:**
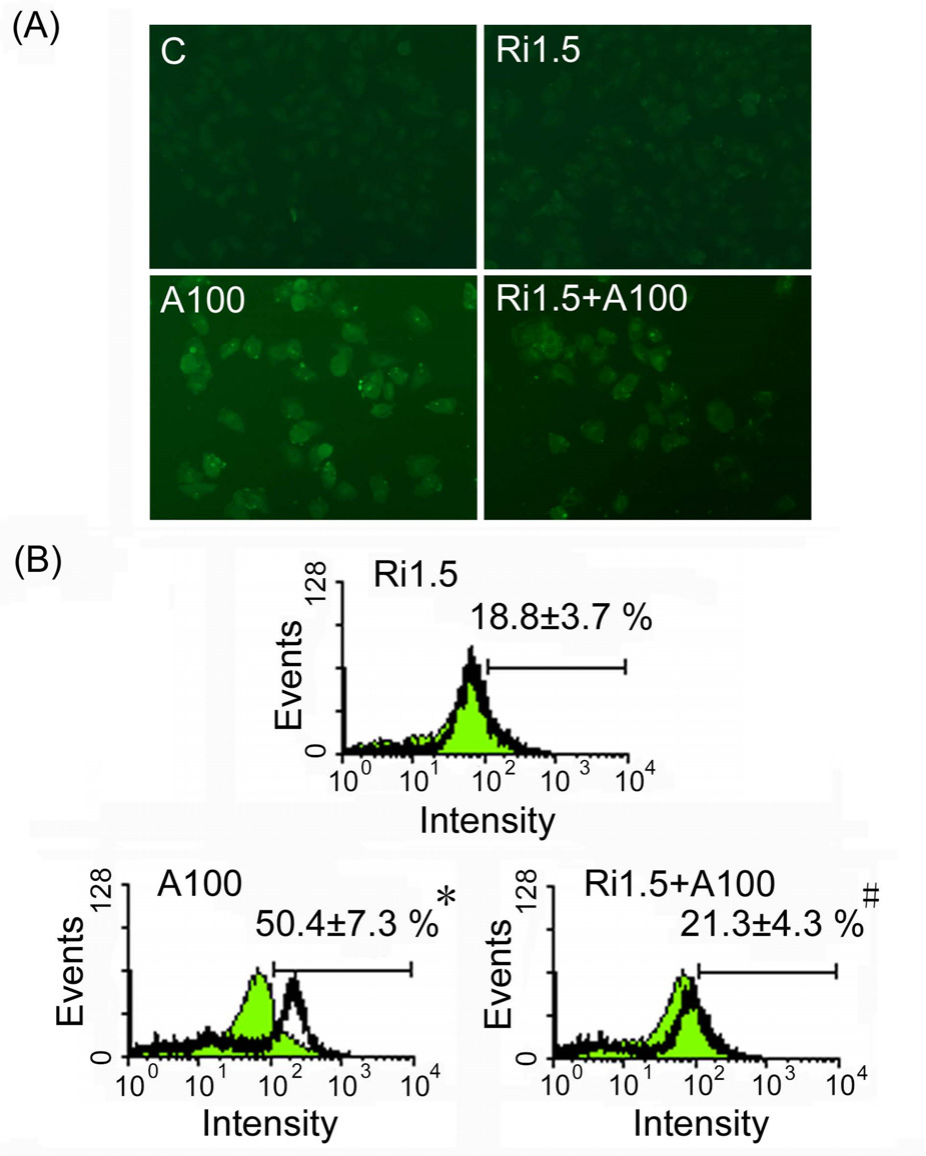
**The RhoA inhibitor, C3 exoenzyme, abolishes arecoline-induced actin stress fiber formation**. (A) HA22T/VGH cells were left untreated (C), treated with 1.5 μg/ml of C3 exoenzyme for 25 h (Ri1.5), with 100 μg/ml of arecoline for 24 h (A100), or with C3 exoenzyme for 1 h, then with 100 μg/ml of arecoline in the continued presence of C3 exoenzyme for 24 h (Ri1.5+A100), then were fixed with paraformaldehyde for actin labeling and observation of stress fiber by fluorescence microscopy (A) or flow cytometry (B). In B, the values are the percentage of actin staining-cells and are the mean ± S.D. for three independent experiments. The green filled area is the untreated control and those delimited by the black lines the treated groups. *: p < 0.05 as compared to the untreated control; #: p < 0.05 as compared to the arecoline (A100) treatment.

**Figure 6:**
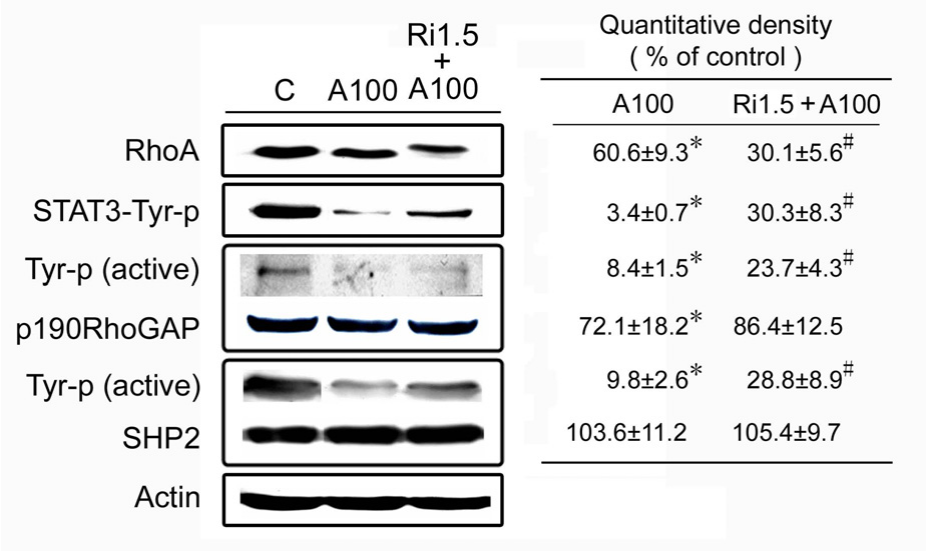
**The RhoA inhibitor attenuates the effects of arecoline on STAT3, p190RhoGAP, and SHP2 in HA22T/VGH cells**. Cells were left untreated or were treated with arecoline alone or arecoline plus C3 exoenzyme as in Fig. 5, then were harvested and proteins were extracted preparing for Western blotting for RhoA, phospho-Tyr705-STAT3 (STAT3-Tyr-p), phospho-Tyr (Tyr-p) p190RhoGAP, and phospho-Tyr (Tyr-p) SHP2, as described in the legend to Fig. 3. β-actin was used as the internal control. The values shown are the quantitative density analysis expressed as the relative density compared to that in untreated cells (control), taken as 100%. The results are expressed as the mean ± S.D. for three separate experiments. *: p < 0.05 as compared to the untreated control; #: p < 0.05 as compared to the arecoline (A100) treatment.

In order to verify the hypothesis that arecoline exerted its effects through the RhoA pathway, we treated HA22T/VGH cells with the RhoA inhibitor C3 exoenzyme for 1 h before, and during arecoline treatment and found that arecoline-induced actin stress fiber formation was reduced (Fig. 5A and 5B) and cell detachment decreased (data not shown). Western blotting (Fig. 6) showed RhoA was inhibited by ADP-ribosylation when C3 exoenzyme was added, as shown by the shift of the RhoA band to a higher molecular weight, whereas the amounts of the active phosphorylated forms of STAT3, p190RhoGAP, and SHP2 were increased. These results demonstrated that all of the effects of arecoline on HA22T/VGH cells were attenuated by addition of the RhoA inhibitor, showing that these effects occurred mainly through the RhoA pathway.

### IL-6 addition has no effect on the arecoline-induced reduction in STAT3 activation

**Figure 7:**
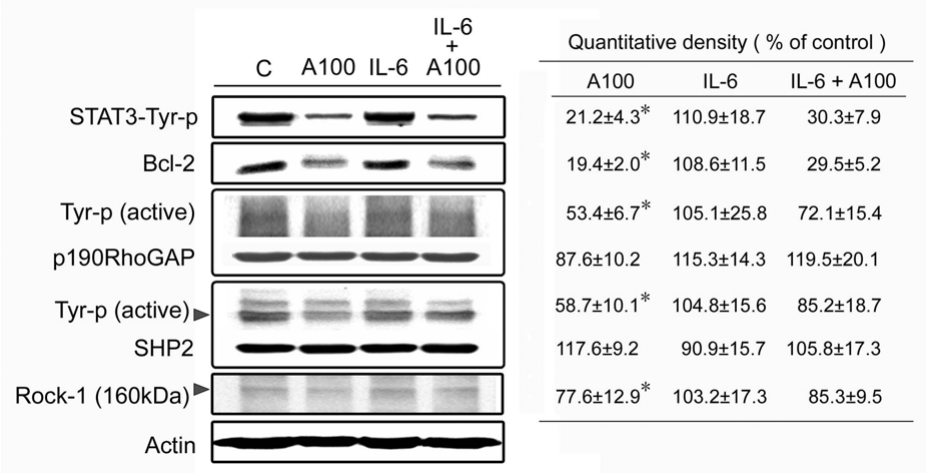
**Addition of IL-6 does not alter the effects of arecoline**. HA22T/VGH cells were left untreated (C) or were incubated with 100 μg/ml of arecoline for 24 h (A100), with 100 ng/ml of IL-6 for 25 h (IL-6), or with IL-6 for 1 h, then with arecoline in the continued presence of IL-6 for 24 h (IL-6+A100). The cells were then harvested and proteins extracted and used for Western blotting for phospho-Tyr705-STAT3 (STAT3-Tyr-p), Bcl-2, phospho-Tyr (Tyr-p) p190RhoGAP, phospho-Tyr (Tyr-p) SHP2, and Rock-1, as described in the legend to Fig. 3. β-actin was used as the internal control. The values shown are the quantitative density analysis expressed as the relative density compared to that in untreated cells (control), taken as 100%. The results are expressed as the mean ± S.D. for three separate experiments. *: p < 0.05 as compared to the untreated control.

To examine whether the arecoline-induced reduction in STAT3 activation could be reversed by addition of IL-6, HA22T/VGH cells were pretreated for 1 h with recombinant IL-6 (100 ng/ml), then co-treated with arecoline for 24 h and proteins were examined by Western blotting. Unexpectedly, as shown in Fig. 7, STAT3 activation was not reversed after IL-6 addition, nor were levels of its downstream effector Bcl-2, total or activated p190RhoGAP, or activated Rock, whereas activated phosphorylated SHP2 levels were increased. These results show that the reduction in STAT3 activation caused by arecoline was not due solely to decreased IL-6 expression and that other pathway(s) might be involved.

### Arecoline interferes with the anchorage-independent growth of HA22T/VGH cells

**Figure 8:**
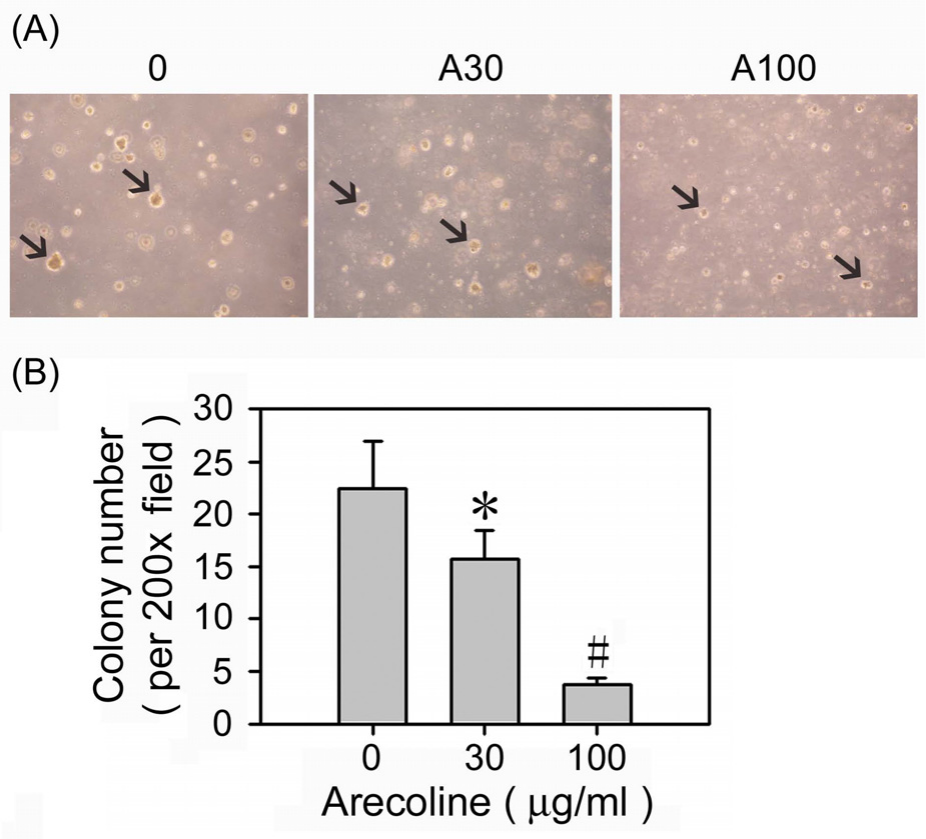
**Arecoline inhibits hepatoma cell colony growth in soft agar**. The growth of HA22T/VGH cells in soft agar was measured in 35 mm diameter dishes with a lower layer of 0.7% agar solution and an upper layer of 0.35% agar solution in which 1 × 10^5 ^cells were resuspended. The soft agar was covered with culture medium alone or containing the indicated concentration of arecoline. After 3 weeks, colonies larger than 0.1 mm in diameter were scored. (A) Representative photomicrographs of soft agar colonies at 200× magnification. The black arrows indicate colonies. (B) Number of colonies in the presence or absence of arecoline. The results are the mean ± S.D. for three independent experiments. *: p < 0.05; #: p < 0.001 as compared to the untreated control.

To examine the effect of arecoline on anchorage-independent growth *in vitro*, we measured the colony formation ability of hepatoma cells in soft agar. Consistent with the above data, the ability of HA22T/VGH to grow in soft agar was markedly inhibited by arecoline (Fig. 8).

## Discussion

**Figure 9:**
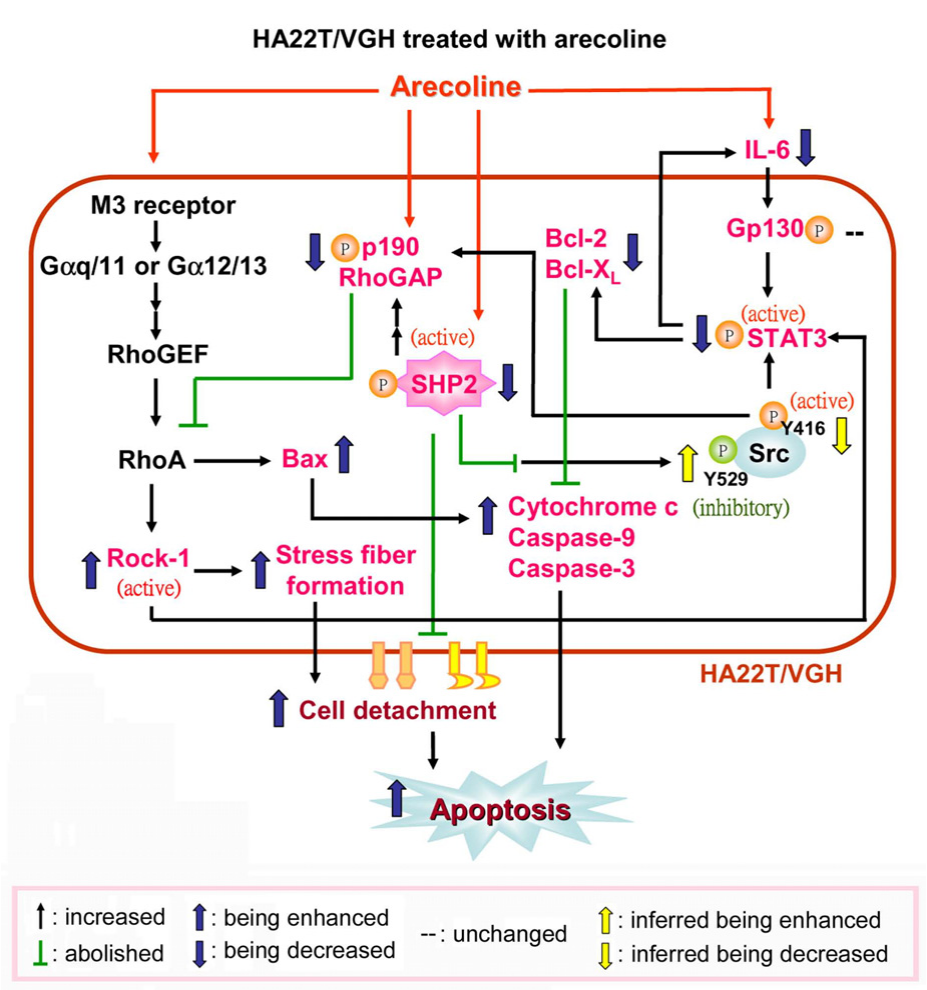
**Schematic representation of the arecoline-stimulated signaling pathways for detachment and apoptosis of HA22T/VGH cells**. Arecoline treatment decreases IL-6 levels, but does not change gp130 of IL-6 receptor. In addition, phosphorylation/activation of STAT3, which provides protection against anoikis, is inhibited and levels of its downstream signals IL-6, Bcl-2, and Bcl-X_L _are decreased, while Bax levels, mitochondrial cytochrome c release, caspase-9 levels, and caspase-3 activity are increased. Phosphorylation/activation of p190RhoGAP, a RhoA inhibitor, and its upstream regulator, SHP2, are inhibited, while the activation/cleavage of Rock-1, a RhoA downstream kinase, and actin stress fiber formation are increased, contributing to anoikis.

In this study, we found that arecoline induced HA22T/VGH hepatoma cells to undergo anoikis. In terms of reducing cell viability, arecoline was effective on HA22T/VGH cells, but not on normal hepatocytes. In HA22T/VGH cells, arecoline caused actin stress fiber formation, resulting in cytoskeletal changes and subsequent apoptosis. Furthermore, IL-6 expression and phosphorylation of its downstream effectors, STAT3, Bcl-2, and Bcl-X_L_, all of which provide protection against anoikis, were decreased. However, the arecoline-induced reduction in STAT3 activation could not be reversed by addition of IL-6. In addition, phosphorylation of p190RhoGAP, a RhoA inhibitor, and of its upstream regulator, SHP2, was decreased and Rock-1, the downstream effector of RhoA, was activated (Fig. 9). These effects were attenuated when a RhoA inhibitor was added, showing that the RhoA pathway was involved in the effects of arecoline on HA22T/VGH cells.

In the liver, STAT3 is mainly activated by IL-6 and related cytokines [15,16]. In the IL-6 signaling pathway, the functional IL-6 receptor complex is composed of an IL-6 receptor that binds IL-6 and a signal transducing receptor component, gp130 [20]. After binding to gp130, STAT3 becomes tyrosine-phosphorylated by JAK kinases, dissociates from the receptor, dimerizes, and translocates to the nucleus [20]. STAT3 has been shown to be constitutively active in a growing number of diverse human cancer cell lines and tumor tissues [13,14], including hepatoma cells [21]. Many proteins that are crucial for tumor cell proliferation and survival have been found to be regulated by STAT3; these include Bcl-2, Bcl-X_L_, cyclin D1/D2 [13,14] and IL-6 [22], which are considered to contribute to the anoikis resistance-inducing property of STAT3. We found that arecoline treatment resulted in a marked reduction in phospho-Tyr705 STAT3 and downregulation of its downstream signaling proteins, IL-6, Bcl-X_L_, and Bcl-2. Interestingly, the reduction in STAT3 activation caused by arecoline was not reversed by addition of IL-6. Thus, we propose that the STAT3 inactivation caused by arecoline does not occur solely through the IL-6 signaling pathway and that other pathway(s) are involved.

Because of its cholinergic properties, arecoline has been used to improve verbal memory in patients with Alzheimer's disease [1,2]. Arecoline exerts its effects mainly via M1, M2, and M3 muscarinic acetylcholine receptors [23-25]. Previous studies have shown that rat hepatocytes express M3 receptors [26]. Further, muscarinic receptor-elicited signals have been shown to affect liver metabolism [26]. Muscarinic receptors are coupled to G proteins. Activation of the G protein-coupled M3 receptor regulates cell growth [27,28], and the involvement of RhoA as a downstream effector in M3 receptor-coupled signals has been established [29]. We therefore suppose that arecoline acts on RhoA activity through M3 receptor-coupled G proteins. In addition, our results showed that the activation/phosphorylation of p190RhoGAP, a RhoA inhibitor, was inhibited by arecoline, which would also enhance RhoA activity.

Apoptosis is characterized by marked morphological changes, including contraction and membrane blebbing. RhoA and its effector Rho-associated kinase (Rock) are involved in the actin-myosin system and have been implicated as the source of the contractile force that drives these morphological changes [19]. A recent study demonstrated that RhoA is involved in tension-dependent death upon cell detachment [8]. RhoA activation increases the cell tension by promoting stress fiber formation, forcing cell detachment and apoptosis. Moreover, a recent report showed that RhoA and Rock also upregulate Bax to activate a mitochondrial death pathway and induce cardiomyocyte apoptosis [30]. Rock is cleaved by caspase during apoptosis to generate a truncated active form [19] and several studies have indicated that Rock activation by caspase-3 seems to be responsible for bleb formation in apoptotic cells [19,31]. Our results clearly showed that Rock-1 cleavage and Bax levels, mitochondrial cytochrome c release, and apoptosis were increased by arecoline treatment and that a RhoA inhibitor attenuated these effects, showing that RhoA/Rock were involved.

Rho family GTPases and STAT3 regulate cell proliferation and gene induction, and their activating mutants are known to be oncogenic [32,33]. Recently, several studies have searched for their functional connection and reported that Rho GTPase can activate STAT3 by induction of Tyr705 phosphorylation, and that activated STAT3 can, in turn, mediate certain aspects of Rho GTPase signaling [34-37]. Debidda *et al. *[37] reported that genetic deletion of STAT3 leads to a loss of response to RhoA in myosin light chain phosphorylation and actin stress fiber induction, but sensitizes cells to RhoA- or Rock-stimulated cell migration. In this study, we found that inhibition of RhoA activation enhanced STAT3 activation in arecoline-treated HA22T/VGH cells, resulting in a reduction in anoikis. These findings suggest that a connection exists between STAT3 and the RhoA/Rock signaling pathway. It is important to clarify how signaling networks are controlled by arecoline through these two important classes of intracellular signal molecules, but there are not enough data in the present study to reveal this complicated link. Further experiments are needed to explore what transcriptional events and target genes that are integral parts of anoikis of the RhoA/Rock signaling paradigm are turned on by activated STAT3.

The HA22T/VGH cells used in this study are a poorly differentiated hepatoma cell line lacking p53 expression [38]. It has been demonstrated that the tumor suppressor p53 can restrict RhoA activation [39,40]. This may mean that, in HA22T/VGH cells, RhoA can be activated easily. In unpublished experiments, we found more activated RhoA and Rock-1 in HA22T/VGH cells than in a wild-type p53 HepG2 hepatoma cell line and showed that HA22T/VGH cells are more sensitive to arecoline-induced anoikis than HepG2 cells. Accordingly, we propose that the p53 deletion in HA22T/VGH cells might contribute to the activation of RhoA/Rock.

Previous studies have shown that SHP2 plays an essential role in integrin signaling. Dominant-negative mutants of SHP2 inhibit integrin-stimulated focal adhesion and stress fiber turnover, cell spreading, and proliferation [12]. SHP2 promotes Src family kinase activation, allowing SHP2 to regulate RhoA activity [18], and may also participate in STAT3-related signaling [41]. We propose that the reduction in SHP2 activation/phosphorylation caused by arecoline might affect RhoA/Rock and STAT3 signal transduction and interfere with integrin signaling and subsequently disrupt the cell-matrix interaction.

Although the cytotoxicity, genotoxic, and mutagenicity of betel nut ingredients, including arecoline, have been extensively investigated [42-44], there is only limited evidence for carcinogenicity of arecoline in experimental animals [45]. In *in vitro *studies, Jeng *et al*. [46,47] found that arecoline significantly inhibits cell proliferation and DNA synthesis in human oral mucosal fibroblasts at concentrations higher than 50 μg/ml, but does not induce DNA strand breaks, even at concentrations of 400 μg/ml. Sundqvist *et al*. [48] found that arecoline does not cause significant DNA single strand breaks even at a concentration of 5 mM in cultured human buccal epithelial cells. Lee *et al*. [49] demonstrated that arecoline induces both cell necrosis and apoptosis in human KB epithelial cells at concentrations of 0.2-1.2 mM. Thus, it seems that arecoline concentrations higher than 0.2 mM may be toxic for normal cells and further investigations are required to determine whether low-dose (less than 0.2 mM or 85 μg/ml) arecoline is toxic. However, this study highlights the possibility that low-dose arecoline might be useful in the treatment of hepatoma and provides clues for studying the arecoline-induced detachment of hepatoma cells.

## Conclusions

We propose that arecoline induces anoikis of HA22T/VGH cells that involves inhibition of STAT3 and increased RhoA/Rock activation and that the STAT3 and RhoA/Rock signaling pathways are connected. Importantly, this study shows that arecoline induces the death of HA22T/VGH hepatoma cells, but not normal hepatocytes. Since arecoline has diverse biological functions, additional studies are needed to evaluate its cytotoxicity for other cells.

## Methods

### Reagents and antibodies

Arecoline hydrobromide (methyl 1-methyl-1,2,5,6-tetrahydronicotinate hydrobromide) was obtained from Sigma-Aldrich (St. Louis, MO, USA); its purity was greater than 99.0%. Collagenase type II was from Worthington Biochemical Corporation (Lakewood, NJ, USA). Percoll was from Amersham Pharmacia Biotech (Uppsala, Sweden). RNase A and the protease inhibitor cocktail were from Sigma-Aldrich (St. Louis, MO, USA). Protein assay reagents were from Bio-Rad Laboratories (Hercules, CA, USA). TRIzol reagent was from Invitrogen Life Technologies (Carlsbad, CA, USA). All other chemicals were of analytical grade and purchased from Sigma-Aldrich (St. Louis, MO, USA). The cell-permeable RhoA inhibitor, C3 exoenzyme, was from Cytoskeleton Inc. (Denver, CO, USA). Recombinant human IL-6 was from Peprotech (Rocky Hill, NJ, USA). Mouse monoclonal antibodies against phospho-tyrosine (phospho-Tyr), phospho-Tyr705-STAT3, SHP2, p190RhoGAP, RhoA, Rock-1, Bcl-2, Bax, procaspase-9, or cytochrome c, rabbit polyclonal antibodies against Bcl-X_L _or gp130, and goat polyclonal antibodies against β-actin were purchased from Santa Cruz Biotechnology (Santa Cruz, CA, USA). Horseradish peroxidase-conjugated anti-mouse, goat, and rabbit IgG antibodies were purchased from BD Pharmingen Inc. (San Diego, CA, USA). R-phycoerythrin (RPE)-conjugated rabbit anti-active caspase-3 polyclonal antibodies, RPE-conjugated mouse anti-human β1-integrin monoclonal antibody and the RPE-conjugated mouse IgG isotype control were purchased from BD Pharmingen Inc. (San Diego, CA, USA). The RPE-conjugated rabbit IgG isotype control was from R&D Systems (Minneapolis, MN, USA). FITC-conjugated swine anti-goat IgG antibodies were from Invitrogen Corporation (Camarillo, CA, USA).

### Cell line, cell culture, and arecoline treatment

HA22T/VGH, a poorly differentiated human hepatoma cell line, was obtained from the Bioresource Collection and Research Center (BCRC) in the Food Industry Research and Development Institute (Hsinchu, Taiwan) and was cultured in Dulbecco's Modified Eagle's Medium (DMEM) (Gibco BRL, Grand Island, NY, USA) containing 10% fetal bovine serum (FBS) (Hyclone, Auckland, NZ), 2 mM L-glutamine (Gibco BRL, Grand Island, NY, USA), 0.1 mM non-essential amino acids (Gibco BRL, Grand Island, NY, USA), 100 units/ml of penicillin, and 100 μg/ml of streptomycin (Gibco BRL, Grand Island, NY, USA) at 37°C in a humidified chamber with 5% CO_2_. To investigate the effects of arecoline, various concentrations of arecoline were added to the culture medium for the indicated time period, then the cells were harvested and analyzed.

### Primary hepatocyte isolation

Hepatocytes were isolated using a previously described method [50]. Sprague-Dawley rats were purchased from BioLASCO Taiwan Co., Ltd. (Charles River Technology, Taipei, Taiwan). This study was performed in accordance with the Guide for the Care and Use of Laboratory Animals of the United States National Institutes of Health. The protocol for animal use was reviewed and approved by the Institutional Animal Care and Use Committee (IACUC) of Kaohsiung Medical University (Approval No. 96137). Livers from newborn Sprague-Dawley rats were mechanically dissociated using a scalpel and the liver pieces incubated in Hank's balanced salt solution (pH 7.4) (HBSS) containing 0.6 mM EGTA and 2% bovine serum albumin (BSA) (Ca^2+^-free) (Sigma-Aldrich, St. Louis, MO, USA) by shaking for 10 min at 37°C followed by a brief wash with HBSS. Hepatocytes were further dissociated from the tissue by shaking for 15 min at 37°C in HBSS (pH 7.4) containing 5 mM CaCl_2 _and 1.5 mg/ml of collagenase type II, then were filtered through a 70 μm nylon cell strainer (BD Falcon, Bedford, MA, USA) to remove cellular aggregates and tissue debris. The cell filtrate was centrifuged at 50 ×***g ***for 10 min and the cell pellet resuspended in DMEM and centrifuged at 800 ×***g ***for 30 min on a discontinuous Percoll gradient comprised of 3 ml of 70% and 6 ml of 30% Percoll. The dissociated cells were stratified and viable hepatocytes were found at the interface between the two Percoll layers. The hepatocyte fraction was collected and the cells plated in flasks in DMEM containing 10% FBS and allowed to attach for 2-3 h at 37°C in a humidified chamber with 5% CO_2_, then were washed with DMEM to remove non-adherent hematopoietic cells. The cells were fed with fresh medium every other day and were split at 80-90% confluence. Experiments were performed on day 7-10 post-isolation.

### Cell morphology

Morphological changes were observed under an inverted phase-contrast microscope (Olympus, Tokyo, Japan). Photographs were taken at 200× magnification.

### Cell viability assay

After arecoline treatment, the cells were harvested and viable cells counted using a dye exclusion technique. The cell suspension was centrifuged at 5 000 ×***g***, the supernatant discarded and the cell pellet resuspended in serum-free medium. One volume of 0.4% Trypan blue (Gibco BRL, Grand Island, NY, USA) was added to one volume of cell suspension, then, after incubation at room temperature for 3 min, cells were counted in a hemocytometer. All counts were done in triplicate.

### Detection of cell surface adhesion molecules

HA22T/VGH cells were harvested and washed with serum-free DMEM, then were suspended in DMEM containing 1% BSA and incubated in the dark at 4°C for 30 min with RPE-conjugated mouse anti-human monoclonal antibody against β1-integrin. After two washed with 1% BSA/phosphate-buffered saline (PBS), the cells were fixed by mixing the cells with 4% paraformaldehyde in PBS, then resuspended in 1% BSA/PBS for flow cytometry analysis. Cell fluorescence was measured using a Coulter Epics XL cytometer (Beckman Coulter, Miami, FL, USA). A control sample incubated with RPE-conjugated normal mouse IgG was run in parallel as a negative control. The data were analyzed using WINMDI software version 2.8 (Scripps Research Institute, La Jolla, CA, USA), a minimum of 1 × 10^4 ^cells per sample being evaluated in each case.

### Apoptosis assay

#### Detection of active caspase-3

Active caspase-3 was detected as described previously [51]. Briefly, cells were pelleted, resuspended in 1 ml of 4% paraformaldehyde, and incubated for 30 min at room temperature. The suspension was then centrifuged, the pellet washed twice with PBS, the cells resuspended in 1 ml of 0.1% Triton X-100 and incubated for 30 min at room temperature, then washed as above. Labeling was performed by addition of 100 μl of PBS containing 5 μl of polyclonal RPE-conjugated rabbit anti-active caspase-3 antibodies, incubation at 37°C for 1 h, washing with PBS, and analysis on a Coulter Epics XL cytometer (Beckman Coulter, Miami, FL, USA). A control sample incubated with RPE-conjugated normal rabbit IgG was run in parallel. The data were analyzed using WINMDI software version 2.8 (Scripps Research Institute, La Jolla, CA, USA), a minimum of 1 × 10^4 ^cells per sample being evaluated in each case.

#### DNA fragmentation assay

Cells (5 × 10^6^) were treated with arecoline for 72 h, then the adherent or detached cells were harvested separately or pooled together for DNA fragmentation analysis as described previously [52]. The cells were pelleted and resuspended in 200 μl of lysis solution [10 mM Tris (pH 8.0), 100 mM NaCl, 25 mM EDTA, 0.5% sodium dodecyl sulfate (SDS), 0.5 mg/ml proteinase K] and incubated for 15 h at 50°C. Nucleic acids were extracted by addition of an equal volume of phenol/chloroform/isoamyl alcohol, centrifugation for 20 min at 10 000 ×***g ***4°C, and harvesting the aqueous (top) layer. DNA were precipitated by addition of a 1/2 volume of 7.5 M ammonium acetate and 2 volumes of 100% ethanol, and recovered by centrifugation at 10 000 ×***g ***4°C for 5 min. After rinsing with 70% ethanol, the DNA was resuspended in TE buffer (10 mM Tris, 1 mM EDTA, pH 8.0) and residual RNA removed by addition of 10 μg/ml of RNase A and incubation at 60°C for 1 h. Samples were resolved on a 1.5% Tris-acetate-EDTA-agarose gel, which was stained with ethidium bromide, and the bands visualized and photographed under short-wave UV.

#### TUNEL assay

Terminal deoxynucleotide transferase-mediated dUTP nick-end labeling (TUNEL) assays were performed using an APO-BrdU™ TUNEL Assay Kit (Molecular Probes, Eugene, OR) according to the manufacturer's protocol. Briefly, the cells were incubated for the indicated time before being trypsinized, washed with PBS, and fixed in 2% paraformaldehyde (pH 7.4) for 15 min. The fixed cells were washed twice in PBS and stored at -20°C in 70% ethanol for 12-18 h prior to performing the TUNEL assay. After removing the 70% ethanol by centrifugation, the cells were washed twice in wash buffer, then incubated at 37°C for 60 min with DNA-labeling solution containing terminal deoxynucleotidyl transferase and BrdUTP. After washing twice with rinse buffer, the cells were resuspended for 30 min in the dark at room temperature in antibody solution containing Alexa Fluor^® ^488-labeled anti-BrdU antibody. Flow cytometric analysis was then performed using a Coulter Epics XL cytometer (Beckman Coulter, Miami, FL, USA) to quantify apoptosis. The data were analyzed using WINMDI software version 2.8 (Scripps Research Institute, La Jolla, CA, USA), a minimum of 1 × 10^4 ^cells per sample being evaluated in each case.

### Protein lysate preparation and Western blotting

Sample preparation and Western blotting procedures were performed as described previously [53]. Briefly, cells were harvested and cytosolic extracts prepared using lysis buffer [20 mM Tris-HCl (pH 7.2), 2 mM EGTA, 5 mM EDTA, 500 μM sodium orthovanadate, 10 mM sodium fluoride, 1% Triton X-100, 0.1% SDS and protease inhibitor cocktail]. Protein concentrations were determined using protein assay reagents. Forty to sixty micrograms of protein lysate was analyzed by SDS-polyacrylamide gel electrophoresis. After transfer of the proteins from the gel to a nitrocellulose membrane (Amersham Pharmacia Biotech, Freiburg, Germany), the membranes were blocked for 1 h at room temperature in PBS with 0.05% Tween 20 (PBS-T) containing 5% nonfat dry milk, then incubated with specific primary antibodies and horseradish peroxidase-conjugated secondary antibodies. For reblotting of other proteins on the same membrane, antibodies were stripped using heated 0.1 M glycine solution (pH 2.0) three times and the membrane washed twice with PBS-T. The immunoreactive bands were visualized using an enhanced chemiluminescence kit (Perkin-Elmer Life Sciences, Boston, MA, USA).

### Stress fiber formation assay

#### Immunofluorescence staining

For actin staining, HA22T/VGH cells were incubated for the indicated time before being washed with PBS, fixed for 10 min at room temperature in 2% paraformaldehyde in PBS, and permeabilized for 10 min at room temperature with 0.5% Triton X-100 in PBS. Filamentous actin was stained for 1 h at 37°C with polyclonal goat anti-actin antibodies in PBS, then for 1 h at 37°C with FITC-conjugated swine anti-goat IgG antibodies. Images were obtained at 200× magnification using a Zeiss Axiovert 200 fluorescence microscope.

#### Flow cytometry analysis

HA22T/VGH cells were incubated for the indicated time before being trypsinized, washed with PBS, fixed for 10 min at room temperature in 2% paraformaldehyde in PBS, and permeabilized for 15 min on ice with 90% methanol. Filamentous actin was then stained as above, and, after washing with PBS, samples were analyzed on a Coulter Epics XL cytometer (Beckman Coulter, Miami, FL, USA). The data were analyzed using WINMDI software version 2.8 (Scripps Research Institute, La Jolla, CA, USA), a minimum of 1 × 10^4 ^cells per sample being evaluated in each case.

### Quantitative real-time PCR analysis

Total RNA was isolated using TRIzol reagent according to the manufacturer's instructions. RNA samples (2 μg) were reverse transcribed using random hexamer primers and M-MLV reverse transcriptase (Promega Corporation, Madison, WI, USA) and the cDNA used for real-time PCR performed on a MiniOpticon™ Real-Time PCR Detection System (Bio-Rad Laboratories, Hercules, CA, USA) using iQ™ SYBR^® ^Green Supermix (Bio-Rad Laboratories, Hercules, CA, USA) following the manufacturer's protocol. The PCR amplification reaction mixture (25 μl) contained 50 ng of cDNA, 12.5 μl of SYBR Green Supermix, and 0.2 μM of the IL-6- or GAPDH-specific primer pair. The optimal primer concentrations were determined in preliminary experiments. PCR primers were designed using Beacon Designer software version 2.0 (Premier Biosoft International, Palo Alto, CA, USA) and their sequences were as follows: IL-6 forward, 5'-TCC TGG TGT TGC CTG CTG-3'; reverse, 5'-TCG TTC TGA AGA GGT GAG TGG-3' and GAPDH forward, 5'-GAC ATC AAG AAG GTG GTG AAG CAG-3'; reverse, 5'-GCG TCA AAG GTG GAG GAG TGG-3'. In order to confirm amplification specificity, the PCR products from each primer pair were subjected to melting curve analysis. The reaction conditions were incubation at 50°C for 2 min and initial denaturation at 95°C for 10 min, followed by 40 cycles of denaturation at 95°C for 20 s and annealing at 60°C for 1 min. After real-time PCR, the temperature was increased from 60 to 95°C at a rate of 0.5°C per second to construct a melting curve. A negative control without cDNA was run in parallel with each assay. Results were collected and analyzed using MJ Opticon Monitor Analysis software version 3.1 (Bio-Rad Laboratories, Hercules, CA, USA). Each reaction mixture was amplified in triplicate and the results calculated based on the ΔΔCt method [54]. The cycle threshold (Ct) value for the IL-6 gene was corrected using the mean Ct value for the GAPDH gene. Relative gene expression was expressed as the fold change (2^-ΔΔCt^) relative to expression in the untreated control.

### Anchorage-independent growth in soft agar

A soft agar assay was performed as described previously [33]. Briefly, growth in soft agar was measured in 35 mm diameter dishes containing a lower layer of 0.7% agar (Bitek agar; Difco Laboratories, Detroit, MI, USA) solution in DMEM containing 10% FBS and 0.1 mM non-essential amino acids overlaid with 0.35% agar solution, also in growth medium, in which 1 × 10^5 ^cells were resuspended. The soft agar was covered with culture medium alone or containing the indicated concentration of arecoline. Colonies were scored 21 days after preparation (colonies larger than ~0.1 mm in diameter were scored as positive). Cells were maintained in DMEM with 10% FBS and 0.1 mM non-essential amino acids.

### Statistical analysis

All data are presented as the mean ± standard deviation (S.D.) for the number of experiments indicated. Other differences between treated and control groups were analyzed using *Student's t-*test. Statistical analyses were performed using SAS version 6.011 (SAS Institute Inc, Cary, NC). A p value < 0.05 was considered statistically significant.

## Competing interests

The authors declare that they have no competing interests.

## Authors' contributions

HLC performed the research, analyzed the data, and drafted the manuscript. SJS and LWH helped in drafting the manuscript. BSH helped with the Western blotting techniques. YCH helped with the normal hepatocyte isolation techniques. TCH performed the flow cytometry study. KLC designed the research, interpreted the data, revised the manuscript, and gave final approval for the version to be published. All authors approved the final version of the manuscript.
